# NaoXinTong Capsules inhibit the development of diabetic nephropathy in *db/db* mice

**DOI:** 10.1038/s41598-018-26746-1

**Published:** 2018-06-14

**Authors:** Shu Yang, Mengyang Liu, Yuanli Chen, Chuanrui Ma, Lipei Liu, Buchang Zhao, Yong Wang, Xiaoju Li, Yan Zhu, Xiumei Gao, Deling Kong, Yajun Duan, Jihong Han, Xiaoxiao Yang

**Affiliations:** 1 College of Biomedical Engineering, Hefei University of Technology, Hefei, China; 2 College of Life Sciences and Key Lab of Bioactive Materials of Ministry of Education, Nankai University, Tianjin, China; 3 Tianjin University of Traditional Chinese Medicine, Tianjin, China; 4 Buchang Pharmaceutical Co. Ltd., Xi’an, China; 5 College of Life Sciences and State Key Laboratory of Medicinal Chemical Biology, Nankai University, Tianjin, China

## Abstract

NaoXinTong Capsule (NXT), a Chinese medicine, is currently used to treat patients with cardiovascular and cerebrovascular diseases. Clinical observations indicate its anti-diabetic functions with unclear mechanisms. Herein, we report the effect of NXT on diabetic nephropathy (DN). Type 2 diabetic *db/db* mice were treated with NXT for 14 weeks. In the course of treatment, NXT reduced diabetes-increased glucose levels and improved renal functions. At the end of treatment, we found that NXT ameliorated serum lipid profiles and other biochemical parameters. In the kidney, NXT inhibited mesangial matrix expansion, expression of vascular endothelial growth factor A, fibronectin, advanced glycation end product and its receptor. Meanwhile, it reduced the diabetes-induced podocyte injury by increasing WT1 and nephrin expression. In addition, NXT inhibited accumulation of extracellular matrix proteins by increasing MMP2/9 expression through inactivation of TGFβ/Smad pathway and CTGF expression. Mechanically, NXT activated insulin signaling pathway by increasing expression of INSR, IRS and FGF21, phosphorylation of Akt and AMPKα in the liver, INSR phosphorylation in the kidney, and FGF21 and GLUT4 expression in adipose tissue and skeletal muscle. Taken together, our study demonstrates that NXT inhibits DN by ameliorating glucose/lipid metabolism, maintaining tissue structure integrity, and correcting diabetes-induced renal dysfunctions.

## Introduction

The type 2 diabetes and the diabetes-induced complications are the major causes of morbidity and mortality for the patients worldwide^1^. Among the diabetic complications, diabetic nephropathy (DN) is the most common microvascular complication. It is also a leading cause for the end-stage of renal diseases^2^. Hyperglycemia, hypertension, dyslipidemia and smoking are the major risk factors for DN development^3^. In addition, males are more prone to develop DN than females^4^.

Hyperglycemia in type 2 diabetes is mainly caused by insulin resistance in the liver where the excess glucose is produced by enhanced gluconeogenesis and glycogen breakdown. Hyperglycemia-activated inflammatory cascade is a major contributor to DN development^5^, because inflammation can induce fibronectin expression and extracellular matrix (ECM) accumulation, and consequently accelerate the progress of glomerulosclerosis and tubulointerstitial fibrosis^6^. Moreover, hyperglycemia activates diacylglycerol-protein kinase C (PKC) pathway^7^, which consequently induces expression of transforming growth factor β (TGFβ) and connective tissue growth factor (CTGF) to enhance ECM accumulation^8,9^.

Advanced glycation end products (AGEs) are molecules with a heterogeneous group formed in the non-enzymatic reactions between sugars and free amino groups of proteins, lipids or/and nucleic acids. AGEs promote renal fibrosis by stimulating renal epithelial cells to release chemokines which can facilitate the recruitment of fibrosis-exacerbating macrophages^10^. Associated with DN development, expression of vascular endothelial growth factor A (VEGFA), a major determinant and regulator of angiogenesis, is activated^11^. The activated VEGFA can induce thickening and distortion of podocyte foot processes, and podocyte injury, thereby accelerating DN progression^12,13^. Interestingly, retinal VEGFA expression can be activated by AGEs indicating AGE-VEGFA pathway can play an important role in DN^14^. Indeed, suppression of VEGFA expression by OPB-9195, a novel AGE inhibitor, inhibits DN in rats^15^.

NaoXinTong Capsule (NXT) is a fine powder mixture containing 11 medicinal herbs [*Astragali Radix* (Huangqi), *Paeoniae Radix Rubra* (Chishao), *Salviae miltiorrhizae Radix et Rhizoma* (Danshen), *Persicae Semen* (Taoren), *Angelicae Sinensis Radix* (Danggui), *Achyranthis bidentatae Radix* (Niuxi), *Chuanxiong Rhizoma* (Chuanxiong), *Spatholobi Stem* (Jixueteng), *Cinnamomi Ranulus* (Guizhi), *Carthami Flos* (Honghua) and *Mori Ramulus* (Sangzhi)], 2 kinds of resin medicines [*Olibanum* (Ruxiang) and *Myrrha* (Moyao)] and 3 kinds of animal medicines [*Scorpio* (Quanxie), *Pheretima* (Dilong) and *Hirudo* (Shuizhi)]^16^. The chemical fingerprints or the quantitative content of major active compounds in NXT have been investigated^16–18^. For instance, in a recent work, Wang *et al*. has identified 16 compounds in NXT with quantitative determination of each. The 16 compounds reported in this study can be classified into the following 6 types: phenolic acids (gallic acid, chlorogenic acid, ferulic acid, 3,5-dicaffeoylqunic acid, 1,5-dicaffeoylqunic acid, rosmarinic acid, lithospermic acid and salvianolic acid B); flavonoids (kaempferol-3-o-rutinoside, calycosin and formononetin); lactones (ligustilide and butyllidephthalide); monoterpenoids (paeoniflorin); phenanthraquinones (cryptotanshinone) and furans (5-hydroxymethylfurfural)^18^.

NXT has been approved by the Sino Food Administration and Drug (SFDA) as a traditional Chinese medicine for treatment of patients with cardiovascular and cerebrovascular diseases^19–23^. Several cardioprotective actions of NXT have been identified by both *in vivo* and *in vitro* studies. For instance, NXT reduces atherosclerosis by inhibiting maturation of dendritic cells in mice, macrophage iNOS expression and NO production^24^. In H9c2 cardiomyocytes, NXT reduces H_2_O_2_-induced oxidative injury^25^. We previously reported that NXT inhibited diabetes-induced retinal vascular abnormalities, and hepatic inflammation induced by a long-term statin treatment^26,27^. Based on the protections of NXT on vascular system above, in this study, we attempted to investigate the effects of NXT on serum glucose levels, lipid profiles and DN development in *db/db* mice, a typical type 2 diabetic animal model.

## Results

### NXT ameliorates serum biochemical parameters in diabetic mice

**Figure 1:**
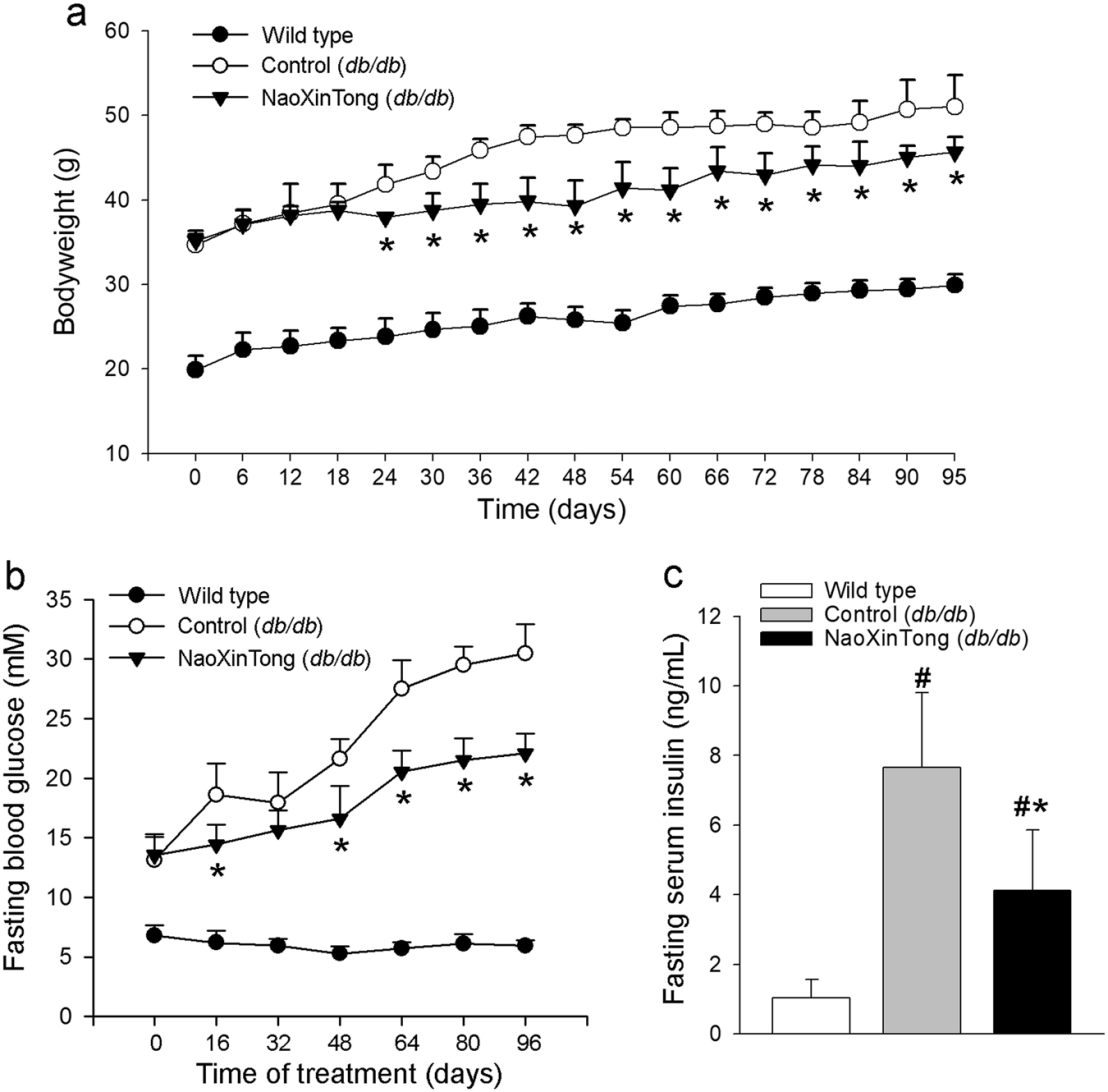
NXT inhibits diabetes-induced hyperglycemia in *db/db* mice. Male *db/db* mice (~6-week old) in two groups (10/group) received following treatment for 14 weeks: Control group, mice were fed normal chow; Naoxintong (NXT) group, mice were fed normal chow containing NXT (620 mpk). Wild type mice fed normal chow were used as the normal control. During the treatment, mouse bodyweight (**a**) and fasting blood glucose levels (**b**) were determined, at the indicated time points of treatment. At the end of study, fasting serum insulin levels (**c**) were also determined. ^*^p < 0.05 *vs*. *db/db* control group; ^#^p < 0.05 *vs*. wild type group (n = 10).

To determine if NXT can inhibit DN, we treated *db/db* mice (~6-week old) with NXT orally for 14 weeks. During the treatment, we routinely determined the bodyweight gain in each group. As shown in Fig. 1a, the average bodyweight of wild type mice was increased from ~20 to ~29 g. In *db/db* control mice, it was increased from ~33 to ~47 g. However, the NXT treatment reduced the rate of bodyweight gain after 3 weeks treatment indicating a moderate inhibition of obesity in *db/db* mice by NXT. Meanwhile, we determined the fasting blood glucose levels once in each duration of 16 days. Compared with the stable blood glucose levels in wild type mice, Fig. 1b shows that the blood glucose levels in *db/db* control mice kept increasing from 13.1 ± 2.0 mM at the beginning of experiment (mice were at ~6-week old) to 30.5 ± 2.5 mM (~2.3-fold) at the end of 14 weeks experiment. In contrast, NXT substantially slowed the increase of glucose levels (22.1 ± 1.7 mM, ~1.6-fold) suggesting that NXT administration improves glucose metabolism. At the end of treatment, we analyzed the fasting blood insulin levels. Similarly, compared with wild type mice, the serum insulin levels were increased ~7.5-fold in *db/db* control mice. However, the elevation was substantially reduced by NXT treatment (~4.0-fold, Fig. 1c).

To determine the effect of NXT on hyperlipidemia in *db/db* mice, serum lipid profiles were analyzed at the end of the study (upper panel, Table 1). Compared with wild type mice, much higher total cholesterol (T-CHO) levels (~1.93-fold) were observed in *db/db* control mice. The increased T-CHO levels were due to increased triglyceride (TG)-rich lipoproteins levels, low-density lipoprotein cholesterol (LDL-CHO, ~3.12-fold) and very low-density lipoprotein cholesterol (VLDL-CHO, ~9.7-fold), particularly the VLDL-CHO levels (Table 1). However, NXT decreased T-CHO, LDL-CHO and VLDL-CHO levels. For instance, both LDL-CHO and VLDL-CHO levels in NXT-treated *db/db* mice were reduced to that of wild type mice (upper panel, Table 1). Taken together, the results in Fig. 1 and Table 1 suggest that NXT ameliorates serum glucose and lipid levels in *db/db* mice.

**Table 1:** NXT ameliorates serum biochemical parameters of *db/db* mice.

Group	Wild type mice	*db/db* mice	
		Control	NaoXinTong
Total cholesterol (mM)	0.907 ± 0.037	1.751 ± 0.311^#^	1.082 ± 0.153*
LDL-cholesterol (mM)	0.097 ± 0.039	0.303 ± 0.090^#^	0.102 ± 0.027*
HDL-cholesterol (mM)	0.738 ± 0.018	0.788 ± 0.176	0.830 ± 0.130
VLDL-cholesterol (mM)	0.070 ± 0.020	0.680 ± 0.056^#^	0.140 ± 0.041*
Triglyceride (mM)	0.232 ± 0.047	0.633 ± 0.147^#^	0.592 ± 0.138*
Urea nitrogen (mM)	1.434 ± 0.243	2.077 ± 0.332^#^	1.790 ± 0.189*
Creatinine (mM)	12.41 ± 3.265	18.11 ± 2.001^#^	15.50 ± 2.898*

Male *db/db* mice (~6-week old) randomly in two groups (10/group) received the treatment as indicated in Fig. 1. At the end of study, the levels of total cholesterol, LDL-cholesterol, HDL-cholesterol, VLDL-cholesterol, triglyceride, urea nitrogen and creatinine in mouse serum were determined. ^#^, *p < 0.05 *vs*. wild type mice and *db/db* control mice (n = 10), respectively.

### NXT inhibits DN by improving glomerular functions in db/db mouse kidneys

**Figure 2:**
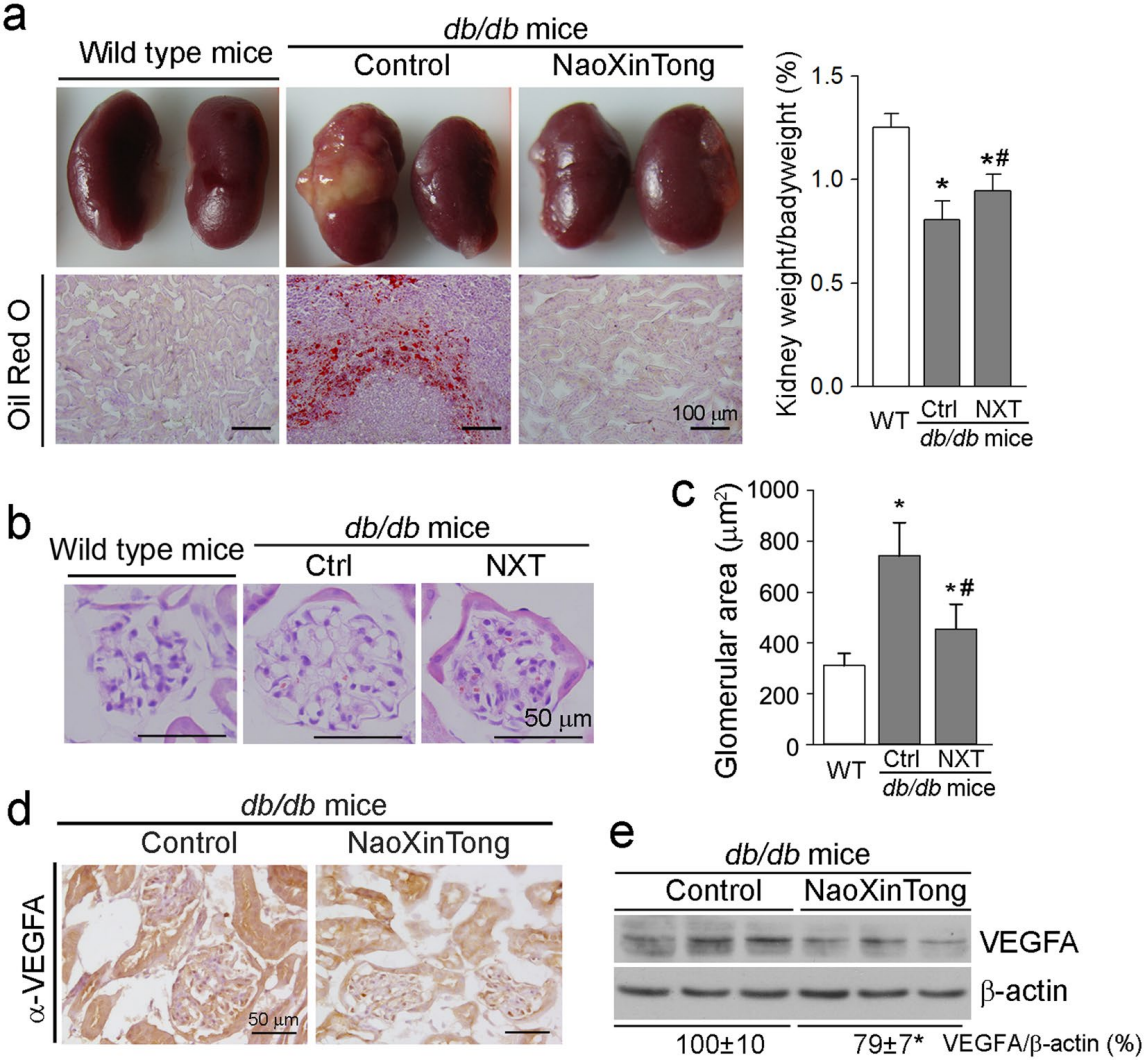
NXT inhibits lipid accumulation, reduces total glomerular area and VEGFA expression in *db/db* mouse kidneys. At the end of treatment as indicated in Fig. 1, mouse kidneys were removed and used to prepare frozen cross sections or total proteins. (**a)** The representative photos of kidneys (upper left panel), ratio of kidney weight to bodyweight (the right panel) and images of Oil Red O staining of cross sections (lower left panel); (**b**,**c)** kidney frozen cross sections were used to detect morphological changes of glomerulus by HE staining (**b**) followed by quantitation of the glomerular area (**c**). ^*^p < 0.05 *vs*. wild type group; ^#^p < 0.05 *vs*. *db/db* control group (n ≥ 5); (**d**,**e)** VEGFA expression was determined by immunohistochemical staining with kidney frozen cross sections (**d**), and by Western blot with total cellular proteins extracted from a piece of kidney (**e**), respectively. *p < 0.05 *vs*. *db/db* control group (n ≥ 5).

At the end of treatment, we observed abnormal kidneys with un-matched size and severe lipid accumulation (upper left panel, Fig. 2a) in some *db/db* control mice (3 of total 10 mice). The renal lipid accumulation was further confirmed by Oil Red O staining kidney cross sections (lower left panel, Fig. 2a). Compared with wild type mice, diabetes also reduced the ratio of kidney weight to bodyweight (right panel, Fig. 2a) indicating the induction of kidney atrophy. However, all the *db/db* mice receiving NXT treatment had normal kidneys without lipid accumulation (left panel, Fig. 2a), and the kidney atrophy was partially corrected (right panel, Fig. 2a).

The renal dysfunction is the consequence of kidney structural abnormalities associated with DN progress, such as glomerular basement membrane (GBM) thickening, mesangial expansion with ECM accumulation, podocyte injury, glomerulosclerosis and tubulointerstitial fibrosis^28^. To determine the effect of NXT on kidney structure, particularly in glomeruli, kidney cross sections were subjected to HE staining followed by quantification of glomerular area. Compared with wild type mice, the glomerular area was clearly increased in *db/db* control mice. However, NXT inhibited the glomerular hypertrophy (Fig. 2b,c).

Antibodies against VEGF can improve hyperfiltration and albuminuria in the diabetic animal models suggesting that VEGF can be a potential therapeutic target for DN^29^. In this study, the results of immunohistochemical staining and Western blot demonstrate that NXT decreased VEGFA expression (Fig. 2d,e).

To determine if NXT treatment can ameliorate renal function related parameters, we initially analyzed urea nitrogen and creatinine levels in mouse serum. As shown in Table 1 (lower panel), compared with wild type mice, both serum urea nitrogen and creatinine levels were increased ~50% in *db/db* control mice but the increases were reduced by NXT treatment. Next, we analyzed levels of nitrogen, creatinine, and microalbumin in urine samples. As shown in Table 2, although NXT slightly affected urinary excretion of nitrogen and creatinine, it significantly decreased excreted microalbumin in urine by ~73 and ~90% at day 45 and 70 or 96 of treatment, respectively. Consequently, the UAlb/UCr levels were substantially reduced by NXT treatment (Table 2). The results of renal function related parameter in both serum and urine indicate that NXT improves glomerular filtration functions.

**Table 2:** NXT reduces urinary microalbumin excretion in *db/db* mice.

Time of treatment (days)	Urea nitrogen (mM)		Urine creatinine (μM)		Urine microalbumin (μg/24 h)		UAlb/UCr (μg/μM)	
	Control	NXT	Control	NXT	Control	NXT	Control	NXT
45	122.6 ± 11.5	128.6 ± 10.7	106.6 ± 18.6	116.5 ± 12.4	229.6 ± 23.1	61.19 ± 4.47^¶^	2.05 ± 0.21	0.47 ± 0.03^¶^
70	115.7 ± 10.0	117.2 ± 8.3	95.6 ± 13.6	97.0 ± 10.0	274.6 ± 20.4	31.3 ± 13.1^¶^	2.21 ± 0.16	0.36 ± 0.05^¶^
96	118.3 ± 9.6	119.5 ± 7.2	93.1 ± 6.6	97.3 ± 3.7	281.2 ± 16.1	29.8 ± 6.91^¶^	2.16 ± 0.12	0.38 ± 0.04^¶^

During the treatment as indicated in Fig. 1, *db/db* mice were placed in metabolic chambers at the indicated time points of treatment to collect urine samples for a 24 h duration. Levels of nitrogen, creatinine and microalbumin excreted in urine were determined, respectively. UCr: urine creatinine; UAlb: urine microalbumin. ^¶^p < 0.05 *vs*. *db/db* control mice (n = 10).

**Figure 3:**
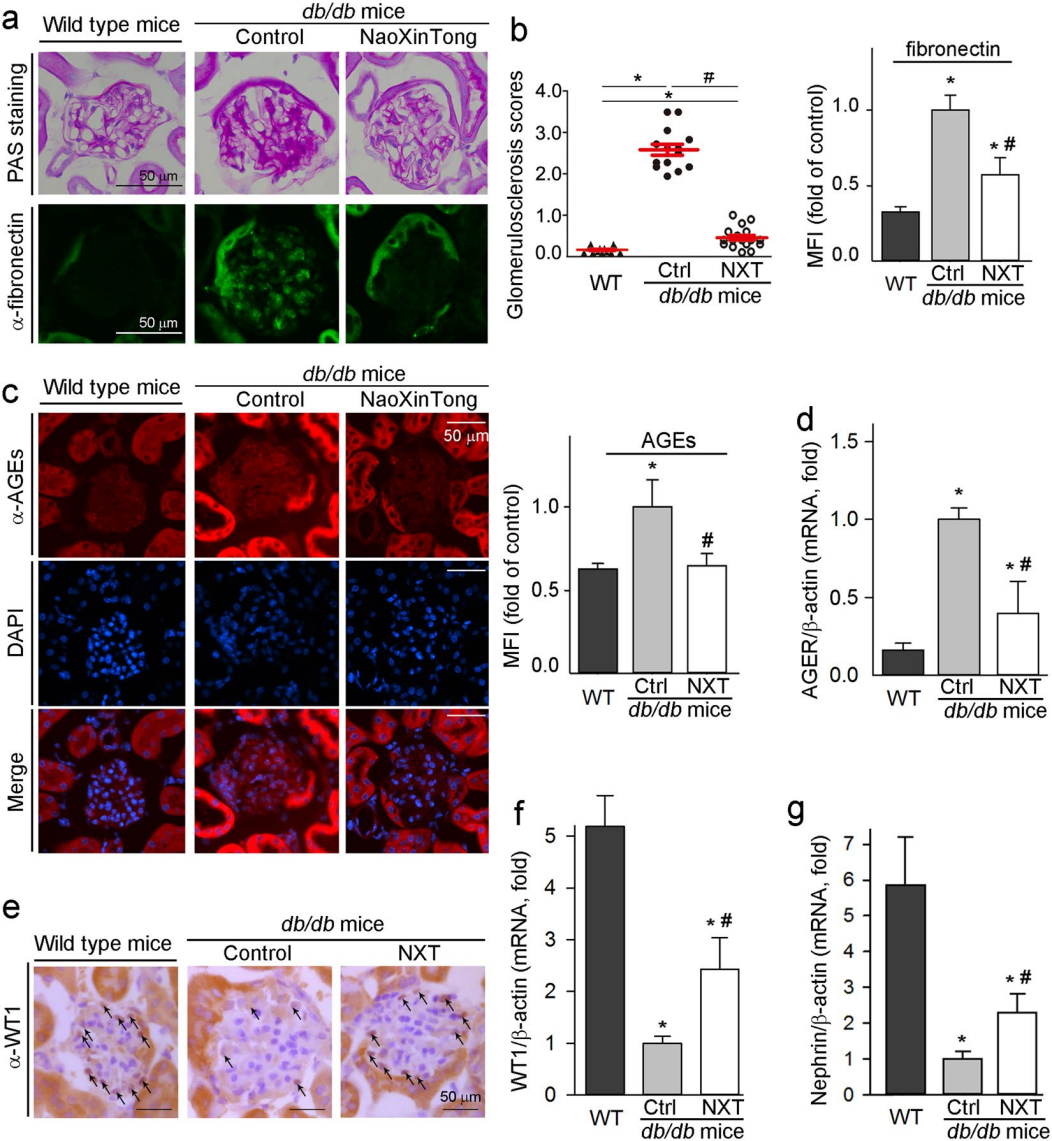
NXT inhibits glomerular mesangial expansion, expression of fibronectin, AGEs and AGER, but activates expression of WT1 and nephrin in *db/db* mouse kidneys. After treatment, kidney cross sections and total RNA were used to complete following assays: (**a)** accumulation of carbohydrate macromolecules and fibronectin expression were determined by PAS staining and immunofluorescent staining, respectively; (**b)** the glomerulosclerosis scores were obtained by calculating the percent of sclerosis area in total area of each glomerulus based on the images of PAS staining (left panel) and the method described in the “Methods”. ^*, #^p < 0.05 (20 glomeruli in each sample were counted). The density of immunofluorescence (mean fluorescence intensity, MFI) of images for fibronectin expression was quantified using segmentation color-threshold analysis method (right panel); (**c**,**d)** expression of AGEs protein or mRNA was determined by immunofluorescent staining (**c**) or qRT-PCR (**d**); (**e**–**g)** expression of WT1 protein (**e**) was determined by immunohistochemical staining; expression of WT1 (**f**) and nephrin (**g**) mRNA was determined by qRT-PCR. ^*^p < 0.05 *vs*. wild type group; ^#^p < 0.05 *vs*. *db/db* control group (n ≥ 5).

High glucose induces fibronectin assembly which can make contribution to collagen IV accumulation, and facilitates uncontrolled ECM accumulation during the DN development^30^. Compared with wild type mice, periodic acid-Schiff (PAS) staining shows the accumulation of carbohydrate macromolecules and elevation of the glomerulosclerosis scores in *db/db* control mice. However, both carbohydrate macromolecule accumulation (upper panel, Fig. 3a) and glomerulosclerosis scores (left panel, Fig. 3b) were reduced by NXT. Fibronectin is a major molecule responsible for accumulation of carbohydrate macromolecules. Correspondingly, fibronectin expression was increased in *db/db* control mouse kidneys, but the increase was substantially reduced by NXT (lower panel, Fig. 3a; right panel, Fig. 3b).

AGE is an important risk factor for DN development. We found that AGE levels in *db/db* control mouse kidney were increased with the majority in tubules, which is in the line that diabetes-induced AGE accumulation is in a tissue or cell type-dependent manner^31^. Interestingly, NXT blocked AGE accumulation in tubules of *db/db* mouse kidney (Fig. 3c). In addition, expression of the receptor for AGE (named as RAGE or AGER) also plays an important role in pathogenesis of DN^32^. Figure 3d shows that NXT significantly decreased diabetes-induced AGER mRNA expression, indicating that NXT can clearly correct the dysfunctions of AGE-RAGE pathway.

Podocytes cover the outer aspect of the GBM and form the final barrier to prevent protein loss. The podocyte injury (e.g., reduction of podocyte number and density per glomerulus) is linked to development of proteinuria and progression of DN in patients. Wilm’s tumor 1 (WT1) protein is a marker of podocytes and plays an important role in maintenance of podocyte function^33,34^. The results of immunohistochemical staining and qRT-PCR analysis show that NXT was able to partially restore diabetes-reduced WT1 protein and mRNA expression (Fig. 3e,f). Nephrin is a key slit diaphragm protein and expressed by podocytes. Nephrin can directly affect insulin signaling *via* modulation of glucose transporters vesicle trafficking at the plasma membrane^35^. Similar to WT1, the decreased nephrin expression in *db/db* mouse kidney was also partially recovered by NXT (Fig. 3g).

### NXT inhibits ECM accumulation in db/db mouse kidney by inactivating TGFβ/Smad pathway and inhibiting CTGF expression

**Figure 4:**
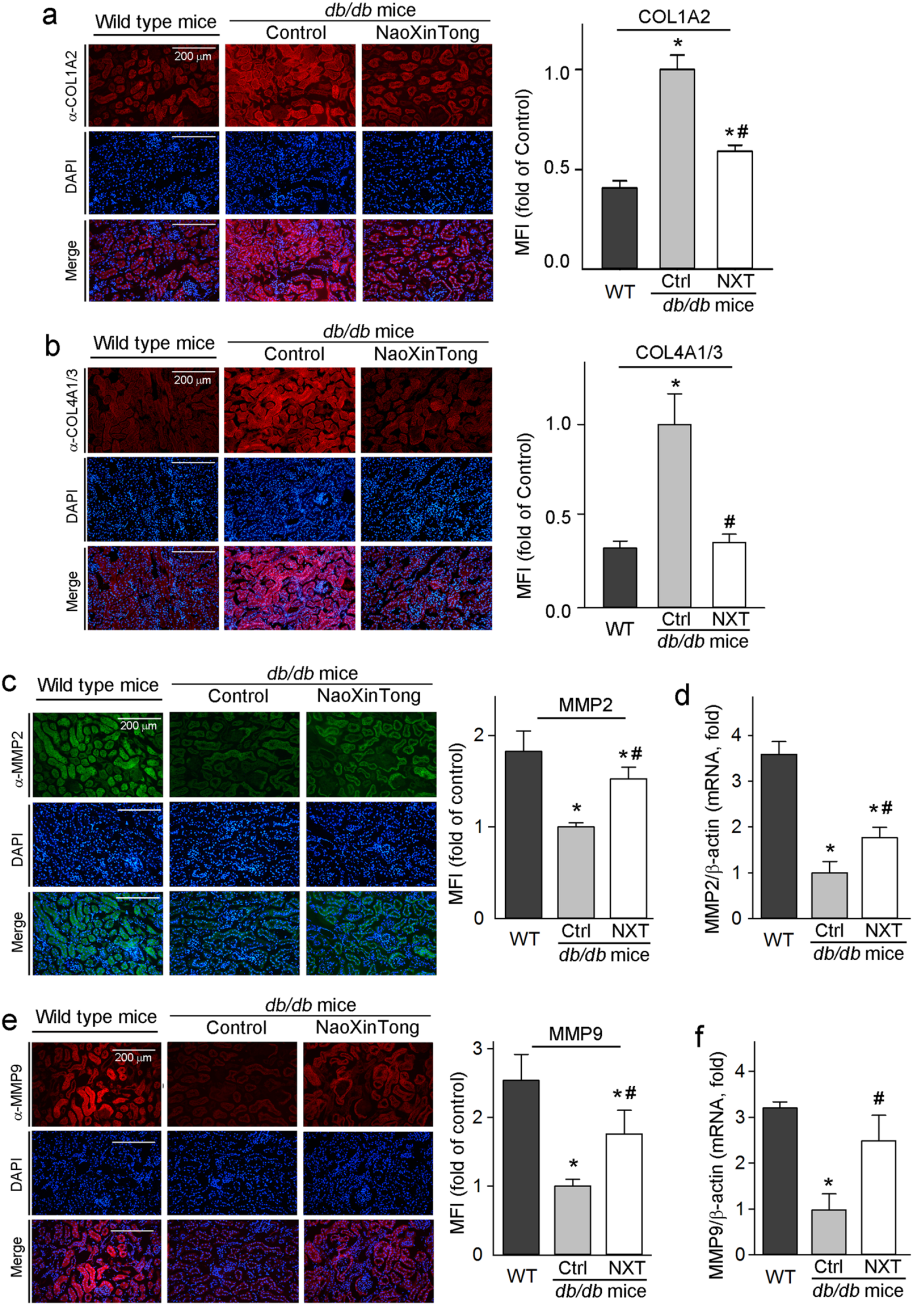
NXT inhibits expression of COL1A2 and COL4A1/3 which is associated with increased MMP2 and MMP9 expression in *db/db* mouse kidneys. Kidney cross sections were used to conduct immunofluorescent staining to determine expression of COL1A2 (**a**), COL4A1/3 (**b**), MMP2 (**c**) and MMP9 (**e**) protein. Total cellular RNA was extracted from a piece of kidney and determined MMP2 (**d**) and MMP9 (**f**) mRNA expression by qRT-PCR. ^*^p < 0.05 *vs*. wild type group; ^#^p < 0.05 *vs*. *db/db* control group (n ≥ 5).

The accumulation of ECM proteins including collagen type I/IV is a main hallmark for DN development^36^. Collagen type IV is a typical collagen of the basement membrane while collagen type I is an important composition of ECM of renal interstitial fibrosis. The results of immunofluorescent staining in Fig. 4a,b indicate that diabetes-increased collagen levels were substantially reduced by NXT. Matrix metalloprotein 2/9 (MMP2/9) catalyze the degradation of ECM components including collagen type IV^36^. By completing immunofluorescent staining and qRT-PCR, we determined that MMP2/9 expression was decreased in *db/db* mouse kidneys, but the decrease was alleviated by NXT (Fig. 4c–f).

**Figure 5:**
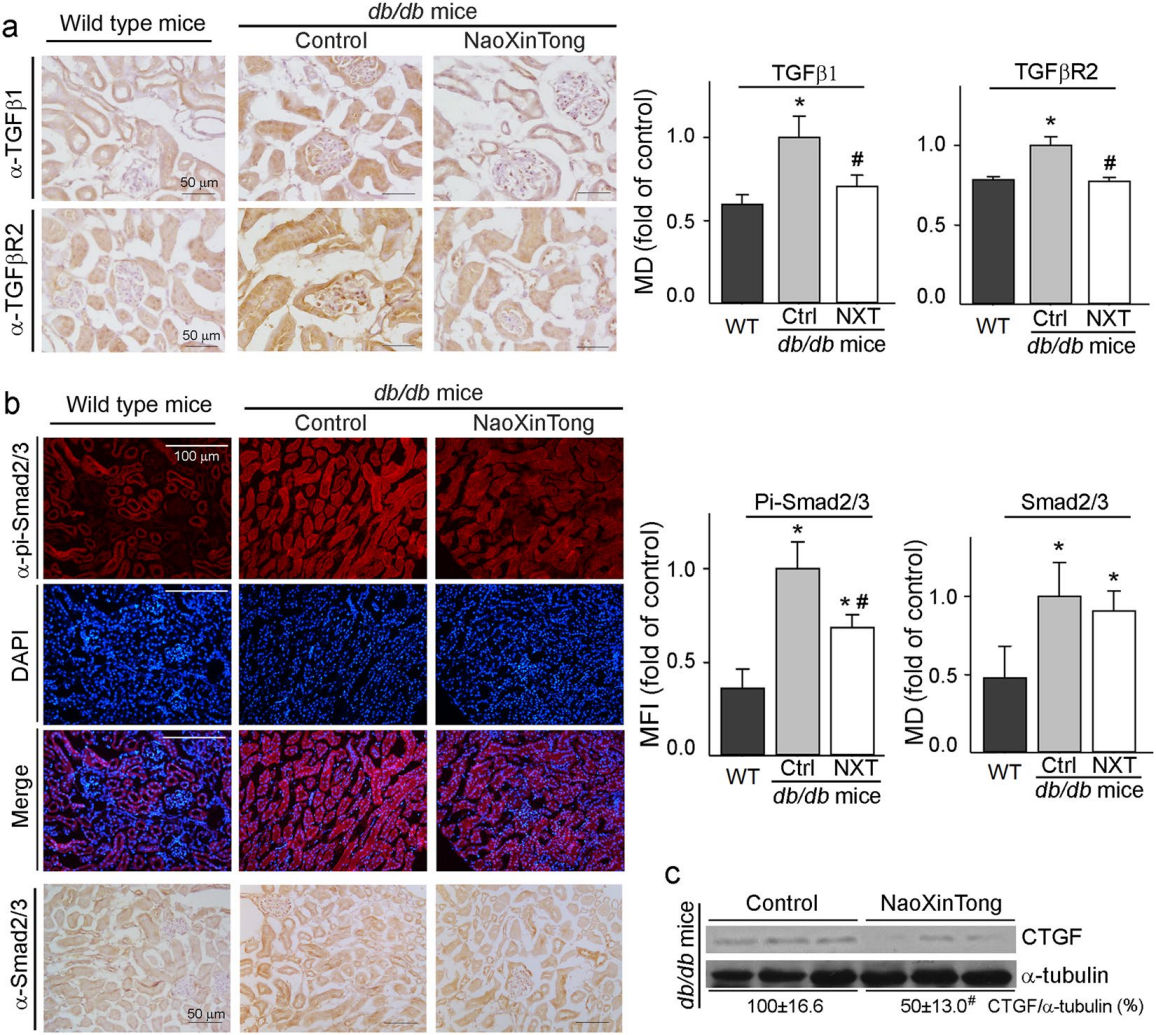
NXT inactivates TGFβ/Smad pathway in *db/db* mouse kidneys. (**a)** Expression of TGFβ1 (upper panel) and TGFβR2 (lower panel) protein in mouse kidneys was determined by immunohistochemical staining; (**b)** expression of pi-Smad2/3 and total Smad2/3 protein in kidneys was determined by immunofluorescent staining or immunohistochemical staining; (**c)** CTGF protein expression was determined by Western blot. *p < 0.05 *vs*. wild type group; ^#^p < 0.05 *vs*. *db/db* control group (n ≥ 5).

Activation of TGFβ/Smad signaling pathway and CTGF expression enhances renal ECM accumulation. We assessed kidney TGFβ1, TGFβ1 receptor II (TGFβR2) and Smad2/3 protein levels by immunohistological staining. Compared with wild type mice, expression of TGFβ1 and TGFβR2 in *db/db* control mouse kidney was increased. However, NXT reduced both to normal levels (Fig. 5a) indicating inactivation of TGFβ/Smad signaling pathway. Consequently, phosphorylated Smad2/3 (pi-Smad2/3) and total Smad2/3 were reduced by NXT with a greater effect on pi-Smad2/3 (Fig. 5b). Meanwhile, compared with *db/db* control mice, NXT substantially decreased renal CTGF protein expression (Fig. 5c).

### NXT activates insulin signaling pathway and improves glucose metabolism

**Figure 6:**
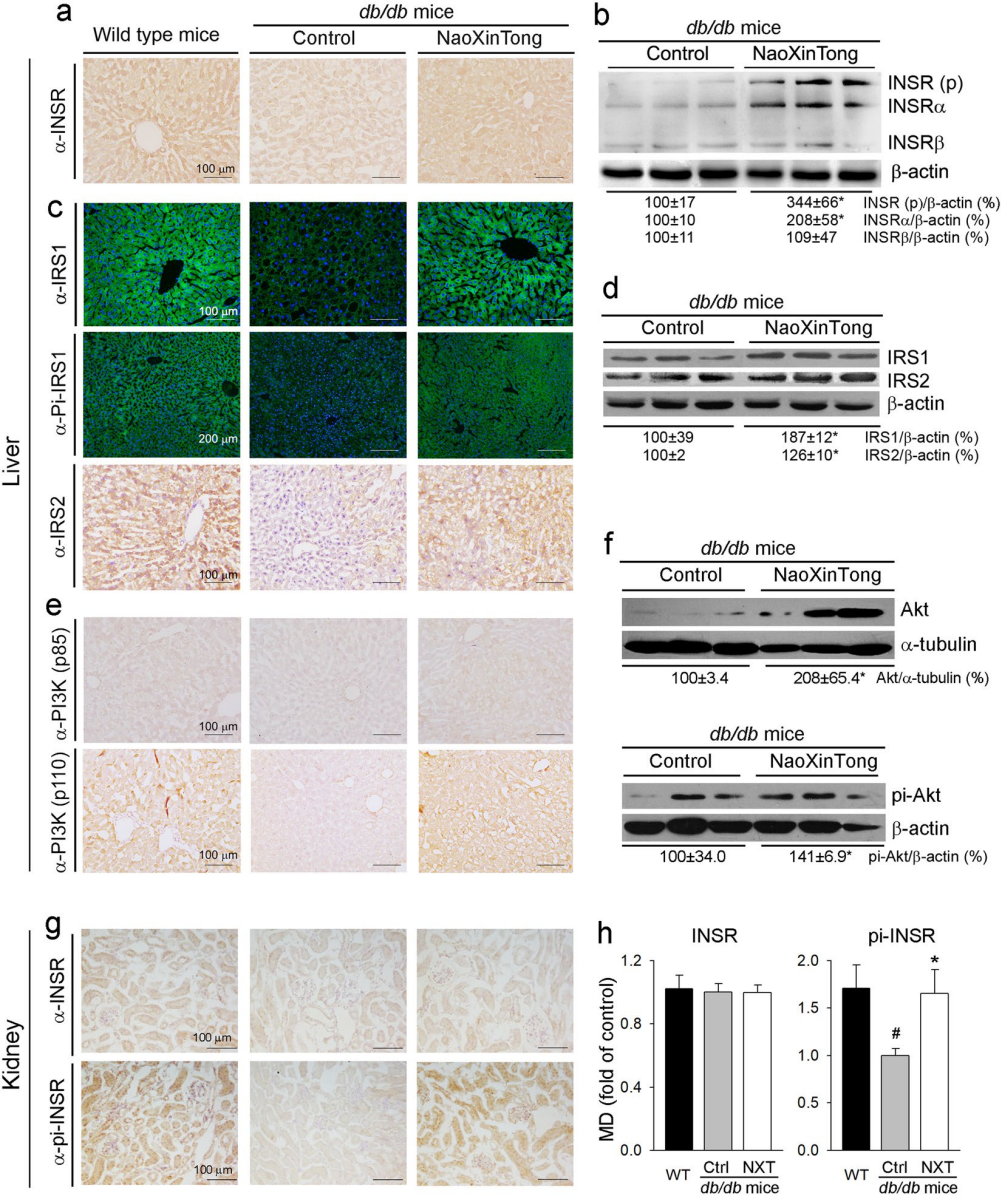
NXT activates insulin signaling pathway in *db/db* mouse liver and kidney. After treatment, mouse liver (**a**–**f**) or kidney (**g**,**h**) samples were used to prepare frozen sections or total cellular proteins. (**a**,**c**,**e**,**g)** expression of INSR, pi-INSR, IRS1, pi-IRS1, IRS2, PI3K (p85) and PI3K (p110) protein was determined by immunohistochemical staining or immunofluorescent staining; (**b**,**d**,**f)** expression of INSR (p), INSRα, INSRβ, IRS1, IRS2, Akt and pi-Akt protein was determined by Western blot; (**h)** the density of immunohistochemical (mean density, MD) of images for INSR and pi-INSR expression was quantified; ^#, *^p < 0.05 *vs*. wild type and *db/db* control group, respectively (n ≥ 5).

To disclose the mechanisms by which NXT inhibits DN, we investigated the effects of NXT on insulin signaling pathway in mouse liver and other tissues. Compared with wild type mice, expression of insulin receptor (INSR) was reduced in *db/db* control mouse liver. However, the reduction was clearly recovered by NXT (Fig. 6a). The results of Western blot analysis confirm that induction of INSR by NXT is mainly contributed by increased INSRα (Fig. 6b). Consequently, the reduced insulin receptor substrate 1/2 (IRS1/2) and phosphorylated IRS1 (pi-IRS1) levels in *db/db* control mouse liver were increased by NXT (Fig. 6c,d). Furthermore, we found that NXT induced expression of both regulatory and catalytic subunit of PI3K, p85 and p110 (Fig. 6e), and consequently the activated PI3K increased both total Akt and phosphorylated Akt (pi-Akt) levels (Fig. 6f).

In mouse kidney, compared with wild type mice, either diabetes or NXT had little effect on INSR expression (upper panel, Fig. 6g; left panel, Fig. 6h). However, the reduced phosphorylated INSR in *db/db* control mice was restored to normal by NXT treatment (lower panel, Fig. 6g; right panel, Fig. 6h). Therefore, Fig. 6 suggests that NXT activates insulin signaling pathway in *db/db* mouse liver and kidney.

**Figure 7:**
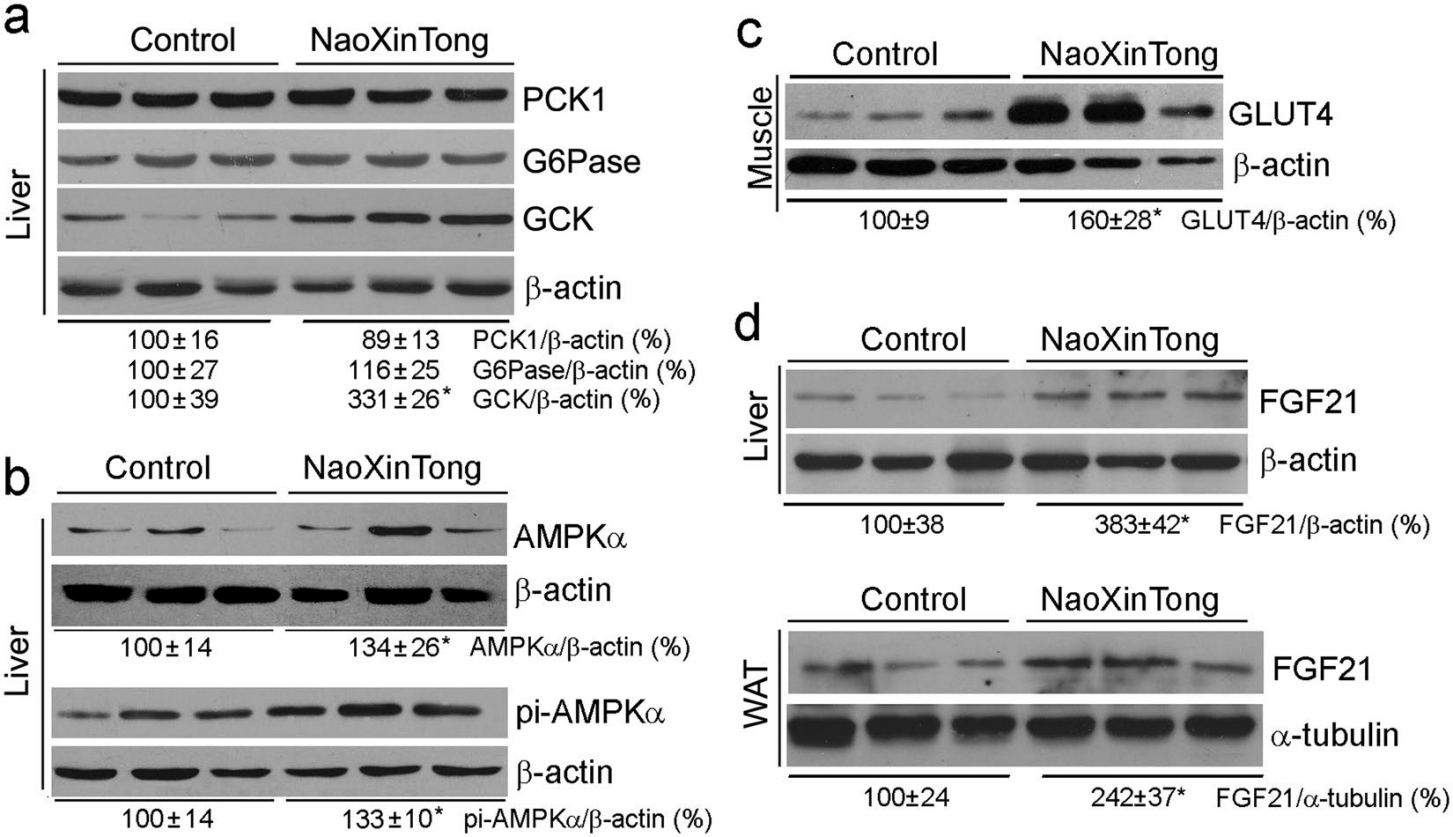
NXT ameliorates glucose metabolism in *db/db* mouse tissues. After treatment, liver, skeleton muscle and white adipose tissue were collected from *db/db* mice followed by total cellular protein extraction. Expression of PCK1, G6Pase, GCK, AMPKα, pi-AMPKα and FGF21 in the liver (**a**,**b**, upper panel of d), GLUT4 in the skeleton muscle (**c**), and FGF21 in the white adipose tissue (lower panel of d) was determined by Western blot. *p < 0.05 *vs*. *db/db* control groups (n ≥ 5).

Activation of glucokinase (GCK) expression can reduce diabetes by enhancing glycogen synthesis and glycolysis. In contrast, phosphoenolpyruvate carboxykinase 1 (PCK1) and glucose-6-phosphatase (G6Pase) have pro-diabetic functions since they are key enzymes for gluconeogenesis. In the liver, NXT had little effect on PCK1 or G6Pase expression indicating that gluconeogenesis is not affected. However, GCK expression was substantially activated (Fig. 7a) suggesting that glycogen synthesis and glycolysis is activated by NXT.

AMPKα can regulate energy metabolism in mammalian cells and be activated by increased ratio of AMP to ATP (AMP/ATP). The activated AMPKα (pi-AMPKα) inhibits fatty acid/cholesterol synthesis and gluconeogenesis, while stimulating fatty acid uptake/oxidation, glucose uptake and mitochondrial biogenesis^37^. Interestingly, we determined that NXT increased both AMPKα and pi-AMPKα levels (Fig. 7b), which suggests that the energy metabolism in *db/db* mice is enhanced. Correspondingly, in the skeletal muscle, NXT increased glucose transporter 4 (GLUT4) expression (Fig. 7c), a molecule responsible for glucose uptake/energy metabolism in the tissue.

Fibroblast growth factor 21 (FGF21) enhances insulin sensitivity and glucose/energy metabolism^38^. Figure 7d shows that NXT increased FGF21 expression in both liver and white adipose tissue of *db/db* mice. Taken together, the results above suggest that NXT protects *db/db* mice against DN by increasing insulin sensitivity and improving glucose and energy metabolism through multiple actions.

## Discussion

DN is one of the diabetic complications with a high morbidity and mortality in patients. Although the current therapeutic interventions can delay the onset and progression of DN, the effects on DN mortality are still limited. Therefore, development of alternative therapeutic approaches is urgent. Traditional Chinese medicine, at least as an adjunctive therapy, has been demonstrated various benefits to patients with different types of diseases. Clinically, NXT is prescribed to patients with cardiovascular and cerebrovascular diseases^19–23^. However, the anti-diabetic effects of NXT have been observed in patients with hyperlipidemia and hyperglycemia. In this study, we treated *db/db* mice with NXT for a long-term and found that NXT clearly inhibited DN development by inhibiting diabetes-induced abnormal kidneys, mesangial expansion, renal accumulation of lipids, AGEs and collagens (Figs 2 and 3). The amelioration of renal functions by NXT is associated with reduction of serum lipid profiles, urea nitrogen and creatinine (Table 1), and excretion of urinary microalbumin (Table 2). The inhibitory effect of NXT on DN is mainly attributed to amelioration of glucose metabolism through activation of insulin signaling pathway in multiple tissues (Figs 1, 6 and 7). Mechanistically, we determined that NXT inhibited TGFβ/Smad signaling pathway and decreased CTGF expression in the kidney which resulted in restoration of diabetes-inhibited MMP2/9 expression (Figs 4 and 5).

Binding of insulin to INSR can activate IRS1 and consequent PI3K/Akt. In *db/db* mice, deficiency of leptin receptor expression results in dysfunction of leptin and inactivation of IRS1^39,40^. Therefore, the animals exhibit severe insulin resistance and hyperglycemia from a very young age (~5-week old). In this study, we determined that NXT increased INSR expression (Fig. 6a,b) which was associated with restoration of IRS1 expression/phosphorylation in *db/db* mouse liver to that in wild type mice (Fig. 6c). NXT also activated IRS1/2 expression (Fig. 6c,d). Consequently, expression of PI3K molecules (p85 and p110) and Akt expression/phosphorylation were increased by NXT (Fig. 6e,f). Although NXT had no effect on INSR expression, it activated INSR by enhancing its phosphorylation in the kidney (Fig. 6g,h). The activation of insulin signaling pathway by NXT results in induction of GCK expression (Fig. 7a). Moreover, both AMPKα expression/phosphorylation in the liver, and FGF21 expression in liver and adipose tissue were activated by NXT (Fig. 7b,d). Taken together, our results demonstrate that NXT controls glycemia by multiple mechanisms in different tissues, mainly by the activation of insulin signaling pathway.

Activation of VEGFA expression and AGE-RAGE pathway as well as their interaction greatly influence DN development. Hyperglycemia induces formation and accumulation of AGE. The consequently activated AGE-RAGE pathway activates reactive oxygen species generation and PKC pathway^41^. Meanwhile, activation of RAGE in podocytes increases VEGFA expression and enhances recruitment/activation of inflammatory cells in diabetic glomeruli which can further accelerate albuminuria and glomerulosclerosis in diabetic kidneys^42^. In animal model, podocyte-specific VEGFA overexpression leads to proteinuria, glomerulomegaly, GBM thickening, mesangial expansion and decreased nephrin^43^. In this study, we observed that administration of NXT inactivated AGE-RAGE pathway, decreased VEGFA protein expression and prevented the podocyte injury by restoring nephrin and WT1 expression in *db/db* mouse kidneys (Figs 2 and 3).

TGFβ plays an important role in tubule glomerular sclerosis in diabetic kidneys by activating matrix synthesis and inhibiting matrix degradation in glomerular mesangial cells, which results in cell proliferation and ECM expansion^44^. In this study, our results show that NXT regulated TGFβ signaling pathway since it decreased TGFβ1 and TGFβR2 protein expression, and correspondingly enhanced MMP2/9 expression in *db/db* mouse kidney. Therefore, inhibition of DN by NXT should be attributed to the blockage of ECM accumulation through inhibition of TGFβ signaling and activation of MMP2/9.

Associated with hyperinsulinemia and hyperglycemia, cholesterol metabolism in *db/db* mice is also exacerbated^45^. Similarly, the impaired lipoprotein metabolism, such as increased VLDL-CHO and LDL-CHO, can be observed in diabetic patients. The diabetes associated with dyslipidemia might be an independent risk factor for DN since the dyslipidemia enhances macrophage infiltration and ECM production in glomeruli^46^. Meanwhile, clinical studies indicate the renoprotective effects of lipid-lowering therapy on diabetic patients^47^. In this study, we determined that NXT restored T-CHO and LDL-CHO levels (upper panel, Table 1), suggesting the lipid-lowering effects of NXT may also make contribution to inhibition of DN. Interestingly, we previously reported that NXT has little effect on lipid profiles in apoE deficient mice^48^ which suggests that improvement of lipid metabolism by NXT in *db/db* mice might be completed by different signaling pathways, such as activation of AMPKα to enhance energy metabolism.

**Figure 8:**
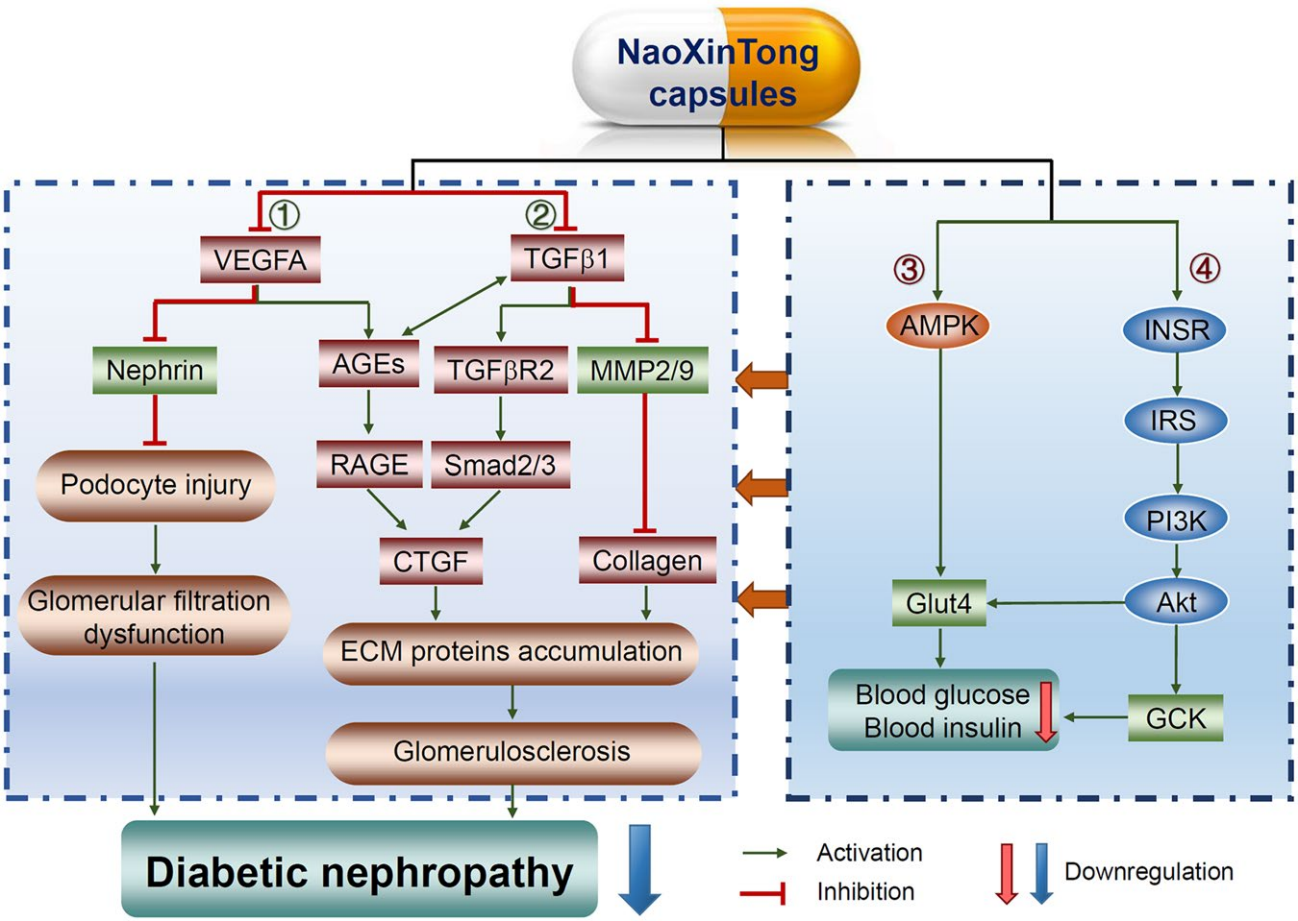
The model depicts the multiple mechanisms by which NXT inhibits the development of diabetic nephropathy in *db/db* mice. Based on the results in this study, NXT treatment inhibits DN in *db/db* mice by regulating the following signaling pathways: ①, ②: direct inhibition of VEGFA and TGFβ1 expression, thereby protecting podocytes from injury and consequently improving glomerular filtration dysfunction; reduction of ECM protein accumulation through inhibition of TGFβ pathway to inhibit glomerulosclerosis; ③, ④: induction of liver GCK expression through activation of Akt pathway and activation of muscle Glut4 which is associated with activation of Akt and AMPK.

In the context of that the effects of current interventions on diabetes are still limited, the alternative strategies including Chinese medicine might offer additional benefits to the patients. Several herbal medicines, such as Tangningtongluo formula, Cistanche tubulosa, Swetia punicea Hemsl and tuberous root of Liriope spicat var., have been demonstrated anti-diabetic effects on patients or animal models^49–52^. In this study, based on the clinical observations, we determined that administration of *db/db* mice with NXT inhibited the development of DN. Furthermore, we presented the results demonstrating that the inhibitory effects of NXT on DN should be attributed to its multiple anti-diabetic actions including improving glucose and lipid metabolism, activating insulin signaling pathway to reduce accumulation of ECM and AGE, and inactivating TGFβ/Smad signal pathway in the kidney (Fig. 8). Our study suggests an important and potential application of NXT for DN treatment.

## Methods

### Materials

NXT was kindly provided by Xianyang Buchang Pharmaceutical Co. Ltd (Shan’xi, China). Rabbit anti-VEGFA, PCK1, GCK, INSR, PI3K (p110), IRS1 and pi-IRS1 polyclonal antibodies were purchased from Proteintech Group (Chicago, IL). Goat anti-AGE polyclonal antibody was purchased from Novus Biologicals (Littleton, CO). Rabbit anti-AKT, pi-AKT, pi-IRS1, IRS2, PI3K (P85), AMPKα and pi-AMPKα polyclonal antibodies were purchased from Cell Signaling Technology Inc (Danvers, MA). The following antibodies were purchased from Santa Cruz Inc.: rabbit anti-MMP2, fibronectin, G6Pase, FGF21, WT1, TGFβ1, TGFβR2 and Smad2/3 polyclonal antibodies; goat anti-MMP9, collagen type I α2 (COL1A2), collagen type IV α1/3 (COL4A1/3), pi-Smad2/3 and CTGF polyclonal antibodies; and mouse anti-GLUT4 monoclonal antibody. The mouse insulin ELISA assay kit was purchased from ABclonal Inc. (Wuhan, China). All other chemicals were purchased from Sigma-Aldrich (St. Louis, MO) except as indicated.

### Animals

The *in vivo* studies with mice were conducted according to the protocol which was granted by the Ethics Committee of Nankai University (Tianjin, China) and conformed to the Guide for the Care and Use of Laboratory Animals published by NIH. Both male type 2 diabetic (BKS.C g-m+/+ Lepr^db^/J, *db/db*, 6-week-old, ~33 g average bodyweight) and C57BLKS/J wild type mice (6-week-old, ~20 g average bodyweight) were purchased from the Animal Center of Nanjing University (Nanjing, China). The animals were maintained at the Animal Center of Nankai University with free access to food and drinking water.

Based on the clinical usage, the dose of NXT to mice could be converted to ~620 mg/day/kg body weight (mpk) or 620 mg/100 g food (mice eat food at ~10% of their body weight daily). Male *db/db* mice at an age of 6-week old were randomly divided into two groups (10 mice/group) and received following treatment: Control group, mice were fed normal chow; NaoXinTong (NXT) group, mice were fed normal chow containing NXT (620 mpk). Male C57BLKS/J wild type mice also at an age of 6-week old were used as a non-diabetic or normal control. The treatment was continued for ~14 weeks. During the treatment, we routinely checked bodyweight, food intake, water drinking and exterior appearance, and did not find difference caused by NXT treatment except that the bodyweight gain was reduced by NXT. At the end of the experiment, all the mice were anesthetized and euthanized by i.p injection of 2,2,2-tribromoethanol (640 mg/kg bodyweight) followed by collection of blood and tissue samples.

### Determination of serum glucose levels

During the treatment, blood glucose levels were determined with an OneTouch glucometer and test strips (LifeScan, Milpitas, CA) using the blood withdrawn from mouse tail vein after overnight fasting, at the indicated time points of treatment.

### Determination of lipid profiles, urea nitrogen and creatinine levels in serum

At the end of experiment, blood samples were collected from mice individually followed by serum preparation. The biochemical parameters in serum samples were determined using a Biochemical Analyzer. These parameters are T-CHO, LDL-CHO, HDL-CHO, VLDL-CHO, TG, urea nitrogen and creatinine.

### Determination of renal functions

At the indicated durations, mice were housed in the metabolic chambers (Nalgene) with free access to food and drinking water for 24 h to collect urine samples. Urinary microalbumin levels were determined with the ELISA assay kit purchased from Elabscience Biotechnology (Wuhan, China). Nitrogen and creatinine levels in urine samples were determined with the assay kits purchased from BioSino Bio-technology and Science Inc. (Beijing, China).

### HE, PAS and Oil Red O staining, and determination of glomerulosclerosis scores

After treatment, the 5 μm cross sections of kidney were prepared and used to conduct the following staining: HE staining for determination of glomerular area; PAS staining for determination of carbohydrate macromolecules; and Oil Red O staining for lipid accumulation^53^.

The images of PAS staining were used to determine the sclerosis area and total area in each glomerulus. The glomerulosclerosis scores were obtained using the method as described^54^ with the following modifications: the percent of sclerosis area in total area of each glomerulus was timed a sclerosis grade factor of, (0) normal glomerulus; (1) sclerotic area ≤25% of the total glomerular area; (2) sclerosis of 25–50% of the total glomerular area; (3) sclerosis of 50–75% of the total glomerular area; and (4) sclerosis ≥75% of the glomerulus. For example, if the percent of sclerosis area in total area of glomerulus in one sample is 26.8%, the glomerulosclerosis for this sample is 26.8% × 2 = 0.54; if the percent of sclerosis area in total area of glomerulus in another sample is 78.9%, the glomerulosclerosis for it is 78.9% × 4 = 3.16.

### Immunofluorescent and immunohistochemical staining

The sections of kidney and liver were subjected to immunofluorescent/ immunohistochemical staining to determine protein expression of fibronectin, AGE, COL1A2, COL4A1/3, MMP2, MMP9, pi-Smad2/3, IRS1, pi-IRS1, INSR and pi-INSR as described^53^. The liver or kidney 5 μm frozen sections were also used to conduct immunohistochemical staining as follows: the slides were rinsed with PBS and incubated in 0.3% H_2_O_2_/PBS solution at room temperature for 10 min. After rinsing with PBS, the sections were blocked with goat serum for 1 h followed by incubation with primary antibody in a humidified chamber for 1 h at room temperature or overnight at 4 °C. After removal of primary antibody by washing with PBS, the sections were incubated with biotin-conjugated goat anti-rabbit IgG for 15 min at room temperature. The sections were then washed with PBS followed by incubation in a HRP-conjugated avidin solution for 15 min before adding the developing solution.

After development, sections were stained with hematoxylin solution for nucleus and then mounted under cover slides with Permount. After adequate drying, the slides were viewed and photographed using a Leica microscope. The density of images was quantified by segmentation color-threshold analysis using morphometry software (IP Lab, Scanalytics, Rockville, MD) as described^55,56^.

### Western blot

A piece of tissue was used to extract total cellular proteins. Protein expression of INSR, INSRα, INSRβ, IRS1, IRS2, Akt, pi-Akt, PCK1, G6Pase, GCK, FGF21, AMPKα and pi-AMPKα in the liver, VEGFA and CTGF in the kidney, GLUT4 in the skeleton muscle, and FGF21 in liver and white adipose tissue were determined by Western blot as described^57^.

### Quantitative real time RT-PCR (qRT-PCR)

After treatment, total RNA was extracted from a piece of kidney followed by cDNA synthesis using a reverse transcription kit (Promega, Madison, WI) and real time PCR with SYBR Green Master Mix (Bio-Rad, Los Angeles, CA) as described^58^. The sequences of primers are listed in Table 3. Expression of AGER, Nephrin, WT1, MMP2, and MMP9 mRNA was normalized with β-actin mRNA in the corresponding samples.

**Table 3:** Sequences of primers for qRT-PCR.

Gene	Forward	Backward
AGER	AGCTATAGGTGCCCTCATCC	ACCTTGACCTGTGCCATCTC
Nephrin	GTCGTAGATTCCCCTTGGGT	GAGAGTCTATGGCCCACCTG
β-actin	CGTTGACATCCGTAAAGACC	AACAGTCCGCCTAGAAGCAC
MMP9	GGTGTGCCCTGGAACTCACACG	AGGGCACTGCAGGAGGTCGT
MMP2	TGGCAAGGTGTGGTGTGCGAC	TCGGGGCCATCAGAGCTCCAG
WT1	CTGTACTGGGCACCACAGAG	CTTAAAGGGAATGGCTGCTG

AGER: advanced glycosylation end product-specific receptor; MMP2/9: matrix metallopeptidase 2/9; WT1: Wilm’s tumor 1.

### Data analysis

All experiments were repeated at least three times, and the representative results are presented. All data were initially subjected to a normal distribution analysis with SPSS software (1-sample K-S of non-parametic test), and the data in normal distribution were then analyzed by the parametric statistics, post hoc test of one-way analysis of variance. A difference was considered to be statistically significant at *p* < 0.05. In addition, all the raw data are available with authors and can be submitted upon request.
